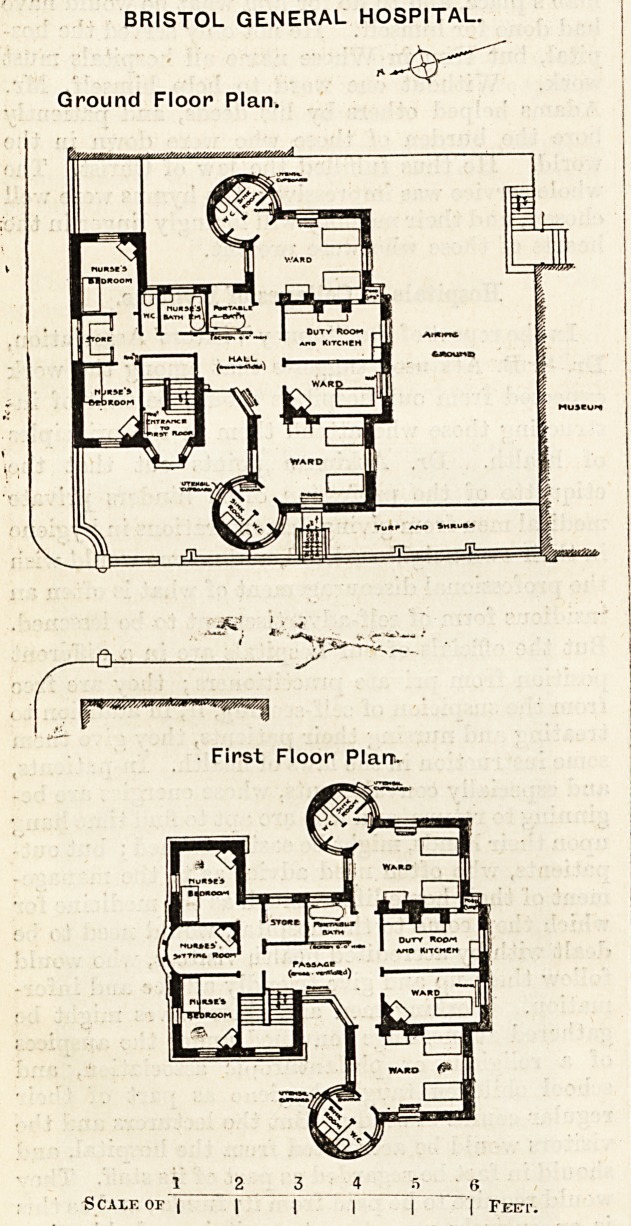# Bristol General Hospital Isolation Wards

**Published:** 1907-04-27

**Authors:** 


### BRISTOL GENERAL HOSPITAL ISOLATION WARDS.

This is a small two-story detached building erected in a courtyard on the northern boundary of the hospital. The accommodation provided on each floor consists of two wards for two beds and one ward for one bed, with a nurses' duty-room. Four bedrooms, a bath-room, and a sitting-room are provided for nurses. To each of the two-bed wards a w.c. and sink-room is provided in circular turret and on each landing is a portable bath.

BRISTOL GENERAL HOSPITAL.

Ground Floor Plan.

First Floor Plan.

The *raison d'être* of a two-bedded ward for isolation purposes is never very apparent, but surely here was an excellent opportunity of applying the principle, now so well-established, of single rooms separated by glass partitions, as exemplified at the Pasteur Hospital, Paris. Five separate rooms opening on to a common corridor, with a nurses' room commanding the whole, would have provided means of isolating five separate diseases. A single sanitary block would have sufficed in place of the two, and the supervision would have been far more effectual than it can possibly be in the three separated rooms. Apart from this criticism, the building is, with one exception, well and carefully planned. The exception we refer to is the form of the sanitary towers. Anything more inconvenient than a circle for this purpose it is difficult to conceive; and we can only suppose that this form was adopted for some architectural reason not apparent from the plan. The architects were Messrs. Oatley and Lawrence, Bristol; the contractor was Mr. R. F. Ridd; and the warming has been carried out by Messrs. J. Crispin and Sons. The cost is not stated.

## Figures and Tables

**Figure f1:**